# Feeding sows resistant starch during gestation and lactation impacts their faecal microbiota and milk composition but shows limited effects on their progeny

**DOI:** 10.1371/journal.pone.0199568

**Published:** 2018-07-03

**Authors:** Julie Leblois, Sébastien Massart, Hélène Soyeurt, Clément Grelet, Frédéric Dehareng, Martine Schroyen, Bing Li, José Wavreille, Jérôme Bindelle, Nadia Everaert

**Affiliations:** 1 Precision Livestock and Nutrition Unit, Gembloux Agro-Bio Tech, TERRA, Teaching and Research Centre, University of Liège, Gembloux, Belgium; 2 Research Foundation for Industry and Agriculture, National Scientific Research Foundation (FRIA-FNRS), Brussels, Belgium; 3 Laboratory of Urban and Integrated PhytoPathology, Gembloux Agro-Bio Tech, TERRA, Teaching and Research Centre, University of Liège, Gembloux, Belgium; 4 Laboratory of Statistics, Informatics and Modelling Applied to Bioengineering, AGROBIOCHEM Department, Teaching and Research Centre (TERRA), Gembloux Agro-Bio Tech, University of Liège, Gembloux, Belgium; 5 Valorisation of Agricultural Products Department, Walloon Agricultural Research Centre, Gembloux, Belgium; 6 Production and Sectors Department, Walloon Agricultural Research Centre, Gembloux, Belgium; University of Illinois, UNITED STATES

## Abstract

**Background:**

Establishment of a beneficial microbiota profile for piglets as early in life as possible is important as it will impact their future health. In the current study, we hypothesized that resistant starch (RS) provided in the maternal diet during gestation and lactation will be fermented in their hindgut, which would favourably modify their milk and/or gut microbiota composition and that it would in turn affect piglets’ microbiota profile and their absorptive and immune abilities.

**Methods:**

In this experiment, 33% of pea starch was used in the diet of gestating and lactating sows and compared to control sows. Their faecal microbiota and milk composition were determined and the colonic microbiota, short-chain fatty acids (SCFA) production and gut health related parameters of the piglets were measured two days before weaning. In addition, their overall performances and post-weaning faecal score were also assessed.

**Results:**

The RS diet modulated the faecal microbiota of the sows during gestation, increasing the *Firmicutes*:*Bacteroidetes* ratio and the relative abundance of beneficial genera like *Bifidobacterium* but these differences disappeared during lactation and maternal diets did not impact the colonic microbiota of their progeny. Milk protein concentration decreased with RS diet and lactose concentration increased within the first weeks of lactation while decreased the week before weaning with the RS diet. No effect of the dietary treatment, on piglets’ bodyweight or diarrhoea frequency post-weaning was observed. Moreover, the intestinal morphology measured as villus height and crypt depths, and the inflammatory cytokines in the intestine of the piglets were not differentially expressed between maternal treatments. Only zonula occludens 1 (ZO-1) was more expressed in the ileum of piglets born from RS sows, suggesting a better closure of the mucosa tight junctions.

**Conclusion:**

Changes in the microbiota transferred from mother to piglets due to the inclusion of RS in the maternal diet are rather limited even though milk composition was affected.

## 1. Introduction

Post-weaning diarrhoea is one of the major health problems in pig husbandry worldwide. It is characterized by a higher risk of infections and a lower feed intake, due to the conversion from milk to solid feed, which has consequences on the gut morphology like the atrophy of the small intestinal villi and hyperplasia of the crypts [1–3]. Weaning troubles are also accompanied with an impairment of the immune function, a higher permeability of the gut mucosa to antigens and lower brush border enzymes activity (lower lactase and sucrase activities), lowering the ability of the piglets to digest feed [1,2,4,5].

Feeding strategies to reduce the risk of post-weaning diarrhoea include the use of prebiotics, probiotics and organic acids in newly weaned piglets’ diet [5]. The mode of action of these feed ingredients relies on their ability to modify favourably the microbiota of the piglets which is very important for their health. Indeed, beneficial bacteria can act as a barrier against pathogens, having the ability to lower the pH of the gastrointestinal tract and produce anti-microbial compounds [6]. Microbiota fermenting indigestible carbohydrates produces SCFA that are an important energy source for the animal and butyrate in particular is a gut health-promoting compound acting as the main energy source for colonocytes and exerting anti-inflammatory properties [7]. It is thus of interest to modify favourably the microbiota towards fermentative butyrate-producing and anti-pathogenic bacteria.

Different moments in the life time of piglets for the feed additive supplementation are currently envisaged in research. The first strategy to favour beneficial bacteria in the gut early in life is to feed the additives to newly weaned piglets to boost their immunity via the development of a beneficial microbiota at weaning. Another strategy is the use of these additives in the sows’ diet in order to promote a rapid colonization of beneficial bacteria and a long-lasting effect for the health of the progeny [8–10]. Several mechanisms are hypothesized concerning the maternal effect. Firstly, acting on the sow’s diet relies on the fact that the microbiota triggering the intestinal immune system in piglets will be acquired from the bacteria present in sows’ faeces, vagina and in milk [8,9]. The purpose then is to modulate the microbiota of the sows to shape a beneficial colonizing microbiota in piglets, improving their immune competence. Secondly, another mechanism that is sought is the modification of the composition of the milk, for nutrients and immunoglobulins (Igs) composition [11], as it has been shown in sows fed a high fibre diet [12] or a diet rich in short-chain fructooligosaccharides [13]. As microbiota impacts the development and maturation of the intestinal immune system [14], and as immunoglobulins act as a first passive immunological defence for piglets [15,16], modifying one or another of these components, or possibly both together, could promote a healthy gut and prepare the piglet for the weaning period.

Resistant starch is the part of starch that escapes enzymatic digestion in the small intestine and can thus be fermented in the colon of the pig [17,18]. It generally comes in ingredients with high amylose contents. Resistant starch can be classified in 5 categories depending on its chemical and physical properties: RS1 (physically inaccessible starch), RS2 (native resistant starch granules), RS3 (retrograded starch), RS4 (starch that has been chemically modified) and RS5 (amylose-lipid complex starch) [18,19]. As a non-digestible but fermentable dietary component, the inclusion of resistant starch in the diet is expected to reduce the energy content of the diet [20], potentially reducing performances and/or feed conversion compared to digestible starch. In turn, it should modulate microbiota composition in the distal small intestine and the large intestine of the animals and subsequently impact fermentation end-products. The production of butyrate is usually specifically increased as starch fermentation is known for being butyrogenic [21,22].

Thus, the purpose of this study was to investigate whether maternal pea starch supplementation could impact the ability of piglets to cope with the weaning period and its associated stresses by comparing the composition of the faecal microbiota and the milk of sows fed two diets contrasting in resistant starch contents. Additionally, the performance, health status and gut immune and morphological status of their progeny was also compared as well as their intestinal microbiota. Pea starch was used as a source of RS because of its ability to produce a high ratio of butyrate during in vitro fermentation [23]; it is considered to be a RS2 type [18,24] and contains 35% of amylose (information provided by the supplier).

## 2. Materials and methods

### 2.1. Animals, diets and housing

All experimental procedures led on sows and piglets were in accordance with European and Belgian regulations concerning laboratory animal welfare. The ethical protocol was reviewed and approved by the Animal Ethical Committee of Liège University (protocol number: 1661). Sows and piglets were housed until weaning at the Walloon Agricultural Research Centre (Gembloux). Landrace sows were inseminated with Piétrain semen and housed in groups on straw litter from one week after artificial insemination (AI) until one week before farrowing. Before the diet change, sows were housed all together in a room that was then divided in two parts to avoid cross contamination after diet change. For farrowing and lactation, they were moved to individual farrowing units, equipped with wood shavings litter, a heat lamp and an extra rear space for sows and piglets accessible by day 5 after delivery. Sows were fed a standard gestation diet until day 88 of gestation, after which they were divided in two dietary groups. The first group (12 sows) received a diet containing 33% of digestible starch (DS diet) and the other group (12 sows) received a diet containing 33% of pea starch (Nastar, Cosucra, Belgium), considered as resistant starch (RS diet). One sow from the RS group had to be removed from the experiment as she had to be treated with antibiotics because of vulva gangrene after delivery. Farrowing was induced by the injection of 2 ml of sodium cloprostenol (92 μg/ml) at 114 days of gestation. Within the DS diet, 4 sows were of 1^st^ parity (P1), 2 sows of 2^nd^ parity (P2), 3 sows of 3^rd^ parity (P3) and 3 sows had a parity higher or equal to 4 (P≥4). Within the RS group, the parities distribution was as follows: 4 P1 sows, 2 P2 sows, 4 P3 sows and 2 P≥4 sows.

Gestation and lactation diets contained 33% of starch, were formulated to be iso-nitrogenous and iso-energetic (net energy) according to NRC requirements (Nutrient Requirements of Swine, 2012). The composition of the diets is shown in Table 1. Between gestation and lactation diets, except for the change of barley into wheat, the same ingredients were used. At weaning (day 28), 44 female piglets (4 piglets/sow) were moved to the Animal Productions Centre in Gembloux and were fed a standard post-weaning diet devoid of antibiotics, prebiotics, probiotics or non-starch polysaccharide (NSP) enzymes. Two littermates were kept together in the same pen and the temperature the day of arrival was maintained at 26°C.

**Table 1:** Composition of sows’ diets during gestation for digestible starch (GDS) and resistant starch (RDS) and during lactation (LDS and LRS) and analyzed chemical composition.

	GDS	GRS	LDS	LRS
Pea Starch	-	33.0	-	33.0
Maize starch	33.0	-	33.0	-
Wheat bran	12.5	12.5	12.4	12.4
Beet pulp	10.1	10.1	6	6
Soy meal	5.1	5.1	13.7	13.7
Sunflower cake	10	10	4.5	4.5
Canola cake	5	5	4	4
Palm cake	4	4	4	4
Wheat	-	-	3.7	3.7
Biscuit flour	3.5	3.5	3.5	3.5
Soy hull	3	3	3	3
DDGS maize	3	3	3	3
Barley	2.6	2.6	-	-
Maize gluten	2	1.8	1.01	0.81
Soy oil	0.3	1.5	1	2.2
Rapeseed flour	1.5	1.5	1.5	1.5
Molasses	1	-	1	-
Limestone	0.94	0.94	1.58	1.58
Fat	0.79	0.79	1.25	1.25
L-lysine	0.37	0.37	0.26	0.26
Salt	0.36	0.36	0.39	0.39
L-thr	0.08	0.08	0.05	0.05
DL-met	0.07	0.07	0.03	0.03
L-try	0.01	0.01	0.05	0.05
Minerals & Vitamins	0.62	0.62	1.102	1.102
**Chemical composition analyzed**^1^				
DM (%)	89.56	90.21	89.80	90.42
OM (%)	84.96	85.43	85.11	84.95
CP (%)	15.37	15.78	16.40	15.05
NDF (%)	22.81	18.26	17.86	17.54
ADF (%)	11.84	9.52	8.68	8.18
EE (%)	2.82	4.53	4.59	5.6
GE (kcal/kg DM)	4009	4110	4049	4112
Total starch (%)	34.4	29.5	31.4	32.8
Resistant starch (%)	0.88	5.41	0.55	8.55

^1^DM: dry matter; OM: organic matter; CP: crude protein; NDF: neutral detergent fibre; ADF: acid detergent fibre; EE: ether extract, GE: gross energy.

### Zootechnical performances

Bodyweight and backfat thickness (Renco lean-meater^®^ of Secrepro, Québec, Canada) of the sows were recorded at days 80 and 107 of gestation and day 28 of lactation to determine the changes between periods. The duration and the piglet expulsion rate of farrowing were determined by recording the time of birth of every piglet.

Piglets were weighed weekly from birth until weaning. After weaning, the diarrhoea status of the piglets was assessed by faecal scoring for 15 days, using a scale going from 0 to 4 (0 = hard pellet, 1 = soft dry pellet, 2 = soft shaped wet pellet, 3 = unshaped soft pellet, 4 = watery). This score was given individually and a mean was calculated for the pen. Piglets were considered to have diarrhoea when the score was 3 or 4. The presence of diarrhoea (score of 3 or 4) was assigned to a “1” value while the absence of diarrhoea (score of 0, 1 or 2) was assigned to a “0” value. The occurrence of diarrhoea was then calculated. As daily recording did not lead to data normality, 3-days data were averaged grouped for analysis. Diarrhoea occurrence was then calculated as the percentage of piglets having a score of 3 or 4 in each pen (0, 50 or 100%) over 3-days periods. The average daily gain (ADG) during the post-weaning period (2 weeks after weaning) was measured by weighing the piglets on a weekly basis.

### 2.2. Feed chemical analyses

Diets were analysed for organic matter (ashing at 550°C for 6h, AOAC 923.03), dry matter (drying at 105°C for 24 h, AOAC 967.03), crude protein (N determination with Kjeltec Analyzer Unit 2300, Foss, Denmark, CP = N×6.25), ether extract (Soxhlet method using ether petroleum, AOAC 920.29), ash-corrected neutral and acid detergent fiber (Fibercap system, Foss, Denmark, Van Soest et al. 1991 [25]) and gross energy (1241 adiabatic bomb calorimeter, PARR Instrument, USA). Starch (total and resistant) was analysed with the enzymatic kit D-Glucose-HK (Megazyme, USA), quantifying glucose concentration after hydrolysis of starch with pancreatic amylase.

### 2.3. Milk

Colostrum was collected within one hour after the birth of the first piglet. Milk samples were collected after the intramuscular injection of 2ml of oxytocin on a weekly basis. Samples were filtered on sterile medical gauze and stored at -20°C until analysis. Protein, lactose and fat contents in milk and colostrum were determined by Fourier transform infrared spectroscopy on a Standard Lactoscope FT-MIR automatic (Delta Instruments, Drachten, The Netherlands). The predictive models provided by the manufacturer were originally designed for cow milk and were consequently adapted for sow milk by a slope and bias correction using a reference set of sow milk for which composition was analysed by standard wet chemistry methods. The R^2^ for each parameter reached 0.99. The IgG and IgA concentrations of colostrum were determined using specific anti-pig antibodies by ELISA (Bethyl Laboratories, Montgomery, USA and R&D Systems, Oxon, UK), following the manufacturer’s recommendations. The plates were read at 450nm on a 96-wells plate reader (Stat-fax 2100, awareness technology Inc, Palm City, USA).

### 2.4. Sampling of intestinal tissues and contents

Faeces were collected directly from the rectum of the sows in sterile bags at day 106 of gestation and day 15 of lactation. They were immediately snap-frozen in liquid nitrogen and stored at -80°C until DNA extraction. Two days before weaning (day 26 of lactation), 16 female piglets (8 DS, 8 RS, 1 piglet/sow) were euthanized by injection of a mix of Xylazine/Zoletil 100 (4 mg of xylazine, 2 mg of zolazepam and 2 mg of tilamine/kg BW) for anaesthesia followed by bleeding. Content from the caecum and the colon as well as tissue from the ileum and colon of the piglets were collected, snap-frozen and stored at -80°C until further analysis. Tissue samples of 5 cm were collected from the duodenum, jejunum and terminal ileum, rinsed with a saline solution and dehydrated in 4% formol prior to long term storage in 70% ethanol. Tissues were then embedded in paraffin, cut with a microtome using Thermo MX35 Ultra blades (Thermo Fisher Scientific, USA) and stained with haematoxylin and eosin. Villus heights and crypt depths were measured on 30 well-oriented couples villus/crypt per animal by 10-fold magnification microscopy (Olympus BX51 Olympus, Japan).

### 2.5. Microbiota composition

DNA was extracted from the sows’ faeces (10 sows/treatment) and piglets colon contents (8 piglets/treatment) using Qiagen QIAamp Stool Minkit (Qiagen, Hilden, Germany), following the manufacturer’s instructions but adding two bead beating steps (FastPrep-24, MP Biomedicals, Illkirsh, France). Quality of DNA was checked on 1% agarose gel and the DNA concentration was assessed by a Nanodrop (Thermo Scientific NanoDrop 2000, USA). DNA was stored at -20°C until sequencing. Sequencing was performed by DNAVision (Gosselies, Belgium), using the Illumina MiSeq (2×300nt) and after amplifying, purifying and tagging the hypervariable regions V3-V4 (Forward primer: 5′-TCGTCGGCAGCGTCAGATGTGTATAAGAGACAGCCTACGGGNGGCWGCAG-3′ and reverse primer: 5′- GTCTCGTGGGCTCGGAGATGTGTATAAGAGACAGGACTACHVGGGTATCTAATCC-3′) following the 16 S Metagenomic Sequencing Library Preparation protocol (Part # 15044223 Rev. B) from Illumina.

### 2.6. Bioinformatics analysis

Raw sequences of 16S rRNA were assigned to each sample, quality checked and trimmed using Basespace default parameters (Illumina). Sequences were assigned to 97% ID OTUs by comparison to the Greengenes reference database 13.8 using the QIIME (Quantitative Insights Into Microbial Ecology) 1.9.0 software. Since samples contained variable number of sequences (62529 ± 4522 for sows, 94139 ± 13830 for piglets), diversity analyses were carried out on samples rarefied at the same sequencing depth (15973 for sows and 53592 for piglets) to avoid bias in sequencing depth between samples. The Beta_diversity_through_plots.py script was used to assess differences in bacterial communities between groups of samples. Beta diversity was visualized using un-weighed and weighed UniFrac distances with Principal Coordinate Analysis (PCoA). The compare_categories.py script, which applied the adonis method on the previously obtained dissimilarity matrices, was used to determine whether communities differed significantly between groups of samples. In addition, the PERMANOVA procedure was performed by period using R studio software (R Studio, Boston, USA), considering the parity (primiparous *vs* multiparous) and the treatment as factors. Multiple_rarefactions.py and alpha_diversity.py scripts were applied to compute alpha diversity metrics, which evaluated diversity within a sample and generated rarefaction curves. Raw sequences have been uploaded in the European Nucleotide Archive database under the project number PRJEB25722.

### 2.7. Short-chain fatty acids determination and calprotectin concentration

Faeces, colon and caecum contents (day 26) were analysed by isocratic HPLC as detailed in Leblois et al. (2017) [26]. Briefly, 1g of sample was diluted in 5g of ultrapure water to reach a 6-fold dilution. Samples were then vortexed for 1 minute to ensure a good solubility and homogeneity of the samples. Aliquots of 2 ml were then centrifuged at 13,000 g, acidified with H_2_SO_4_ and filtered at 0.22 μm. Samples were then analysed for SCFA concentration on a Waters system equipped with an Aminex HPX-87H column (Bio-Rad, Hercules, CA, USA) combined with a UV detector (210nm) at 58°C. The mobile phase was H_2_SO_4_ 5mM. Peaks were integrated with Empower 3 software (Waters, Milford, USA) after the encoding of a standard curve. Results are expressed as mmol.g^-1^ and molar ratios, taking into account the initial dilution. Calprotectin concentration in the colon contents of the piglets was assessed using Porcine Calprotectin *ELISA* Kit (MyBioSource, San Diego, USA) following the manufacturer’s recommendations. Absorbance was measured at 450nm.

### 2.8. Gene expression analysis

RNA was extracted from frozen ileum and colon tissue (day 26) using ReliaPrep^™^ RNA Tissue Miniprep System kit (Promega, Madison, USA). RNA concentration was determined with a Nanodrop (Thermo Scientific NanoDrop 2000, USA) and integrity was checked on a 1% agarose gel. Then, 2 μg of RNA were converted to single-stranded cDNA using GoScript^™^ Reverse Transcription Mix (Promega, Madison, USA), following the manufacturer’s instructions. Specific regions of cDNA coding for housekeeping genes—*glyceraldehyde-3-phosphate dehydrogenase* (*GAPDH*) and *beta actin* (*ACTB*)- , tight junction proteins -*zonula occludens-protein 1* (*ZO-1*) and *Occludin* (*OCLN*)- and proteins involved in the inflammatory response—*tumor necrosis factor alpha* (*TNF-α*), *interleukin 6* (*IL-6*), *nuclear factor kappa B* (*NF-κB*), *transforming growth factor beta* (*TGFβ*), *interferon gamma* (*IFNγ*), *interleukin 1 beta* (*IL-1β*) and *interleukin 10* (*IL-10*)- were then amplified with qPCR (StepOne Plus, Thermo Fisher Scientific, USA) using SYBR Premix Ex Taq II (TakaraBio). Primers and their reference are shown in Table 2. QPCR conditions were optimized to obtain primer efficiency values between 90 and 110% (denaturation at 95°C for 5s, annealing at 60°C for 30s and elongation at 72°C for 30s) and primers specificity was verified through melting curves. GAPDH and ACTB were used as reference genes and were selected after verification of their stability for all experimental conditions.; gene expression was normalized using the 2^-ΔΔCt^ method setting the value of the DS pigs to 1 to allow comparisons.

**Table 2:** Primers used for gene expression analysis.

Primer		Sequence (5’->3’)	Reference	Accession number
*ACTB*	*F*	GGA-CTT-CGA-GCA-GGA-GAT-GG	[27]	XM_021086047
	*R*	GCA-CCG-TGT-TGG-CGT-AGA-GG		
*GAPDH*	*F*	CAT-CCA-TGA-CAA-CTT-CGG-CA	[28]	NM_001206359.1
	*R*	GCA-TGG-ACT-GTG-GTC-ATG-AGT-C		
*TNF-α*	*F*	ACT-GCA-CTT-CGA-GGT-TAT-CGG	[29]	NM_214022.1
	*R*	GGC-GAC-GGG-CTT-ATC-TGA		
*IL-6*	*F*	AGA-CAA-AGC-CAC-CAC-CCC-TAA	[30]	NM_214399
	*R*	CTC-GTT-CTG-TGA-CTG-CAG-CTT-ATC		
*TGFβ*	*F*	GAA-GCG-CAT-CGA-GGC-CAT-TC	[31]	NM_214015
	*R*	GGC-TCC-GGT-TCG-ACA-CTT-TC		
*IFNγ*	*F*	TGG-TAG-CTC-TGG-GAA-ACT-GAA-TG	[32]	NM_213948
	*R*	GGC-TTT-GCG-CTG-GAT-CTG		
*NF-κB*	*F*	CCT-CCA-CAA-GGC-AGC-AAA-TAG	[33]	ENSSSCT00000033438
	*R*	TCC-ACA-CCG-CTG-TCA-CAG-A		
*IL-1β*	*F*	ATG-CTG-AAG-GCT-CTC-CAC-CTC	[34]	NM_214055
	*R*	TTG-TTG-CTA-TCA-TCT-CCT-TGC-AC		
*IL-10*	*F*	CTG-CCT-CCC-ACT-TTC-TCT-TG	[35]	NM_214041
	*R*	TCA-AAG-GGG-CTC-CCT-AGT-TT		
*ZO-1*	*F*	TGA-GAG-CCA-ACC-ATG-TCT-TGA-A	[30]	XM_021098856
	*R*	CTC-AGA-CCC-GGC-TCT-CTG-TCT		
*OCLN*	*F*	CTA-CTC-GTC-CAA-CGG-GAA-AG	[36]	NP_001157119.1
	*R*	ACG-CCT-CCA-AGT-TAC-CAC-TG		

### 2.9. Statistical analyses

All statistical analyses were performed on SAS 9.2 (SAS Inc, USA). Gut morphology was analysed with the NESTED procedure of SAS, with the treatment as fixed class factor and piglet as random factor; piglets’ bodyweight until weaning was determined with the mixed procedure of SAS, time being a repeated effect, sow(treatment) being a random effect and treatment and parity being fixed factors. Milk composition and sows’ performances were analysed with the repeated MIXED procedure of SAS; treatment and parity were used as fixed effects and time was used as a repeated factor. Duration of farrowing and expulsion rate were determined with the MIXED procedure of SAS, using parity and treatment as fixed effects. Calprotectin, gene expression and SCFA were analysed with the MIXED procedure of SAS, using the maternal treatment as fixed factor. Diarrhoea score, piglets’ bodyweight and average daily gain (ADG) post-weaning were analysed with the repeated MIXED procedure of SAS, with time as repeated factor and maternal treatment as fixed effect. Microbiota results were analysed with the non-parametric Kruskall-Wallis test added by Benjamini-Hochberg correction; maternal treatment was included in this test as fixed effect. Pearson’s correlations were determined between the abundance of *Lactobacillus* and of other genera with a relative abundance of >1% of the total microbiota using the proc CORR of SAS. P-values <0.05 were considered as significant. For microbiota analysis, a 0.05<p<0.10 was considered as a trend.

## 3. Results

### 3.1. Zootechnical parameters

No differences between treatments were observed concerning the duration (250 ± 27 min for DS vs 243 ± 53 min for RS, p = 0.95) or expulsion rate (one piglet every 19±2 and 20±5 min for the DS and RS groups, p = 0.63) of farrowing. Changes in bodyweight and backfat thickness between periods were not affected by the dietary treatment either (S1 Table). The survival of piglets until weaning was not affected by the treatment (86.5% of piglets for DS vs 84.7% for RS, p = 0.82). No impact of the maternal treatment or the sex was observed for piglets’ bodyweight until weaning (S1 Fig). None of these parameters were impacted by the parity of the sow.

### 3.2. Colostrum and milk

Globally, lower milk protein concentrations were observed in the RS sows than in the DS group (p = 0.02). An interaction between the treatment and sow parity was observed (see Table 3, p<0.05), showing that for P3 sows, the milk protein percentage was lower at every time point for RS sows (Table 4). Fat percentage was not affected by the RS diet, but an interaction between parity and time was significant (p<0.05). Only in colostrum, parity influenced the fat concentration as the colostrum of first parity sows was richer in fat and then gradually decreased with parity. Milk lactose percentage was not impacted by the treatment (p = 0.09) and a significant interaction between time and treatment was observed (p = 0.01). For colostrum and milk samples collected during week 1, RS sows secreted more lactose (p<0.05) in their milk while this concentration was lower during the last week of gestation (p<0.05). Milk composition changed with time, as protein concentration decreased over time while lactose and fat increased (Table 4).

**Table 3:** P-values of the treatment, time, parity and interactions for protein, fat and lactose content of the milk.

	Protein	Fat	Lactose
Treatment	**0.02**	0.74	0.14
Time	**<0.001**	**<0.001**	**<0.001**
Parity	0.37	0.07	0.30
Treatment*Time	0.11	0.11	**0.01**
Treatment*parity	**0.02**	0.13	0.13
Time*parity	0.43	**0.02**	0.59
Treatment*Time*parity	0.43	0.32	0.93

**Table 4:** Composition (protein, fat and lactose) of colostrum and milk of sows fed digestible starch (DS) and resistant starch (RS) in function of parity. N = 12 for the DS group and N = 11 for the RS group after because of removal of one sow from the experiment.

	Diet	Parity	Time			
			Colostrum	Week 1	Week 2	Week 3
Protein (%)	DS	P1	18.46	6.25	5.63	5.59
		P2	18.61	6.32	5.39	5.16
		P3	19.76	6.42	6.29	6.78
		P≥4	18.83	7.52	5.88	5.60
	RS	P1	17.35	6.09	5.76	5.73
		P2	18.36	6.01	5.80	5.97
		P3	17.33	5.79	5.41	5.69
		P≥4	17.99	5.85	5.89	5.63
	Global SEM		0.33	0.16	0.1	0.14
Fat (%)	DS	P1	9.22	8.90	9.29	8.57
		P2	7.82	8.37	8.72	7.14
		P3	7.78	8.43	9.87	10.46
		P≥4	5.34	8.96	8.46	7.62
	RS	P1	8.76	8.69	9.70	9.94
		P2	8.39	9.17	8.38	9.54
		P3	6.94	8.03	7.55	9.01
		P≥4	6.01	8.95	8.48	8.74
	Global SEM		0.37	0.31	0.28	0.35
Lactose	DS	P1	2.81	4.75	4.96	4.91
		P2	2.75	4.92	5.13	5.17
		P3	2.67	4.75	4.86	4.99
		P≥4	2.69	4.88	5.02	5.18
	RS	P1	2.88	4.89	4.89	4.86
		P2	2.83	4.88	5.13	4.86
		P3	3.07	4.98	5.10	4.95
		P≥4	2.97	4.98	5.02	5.03
	Global SEM		0.05	0.03	0.04	0.03

**Fig 1:**
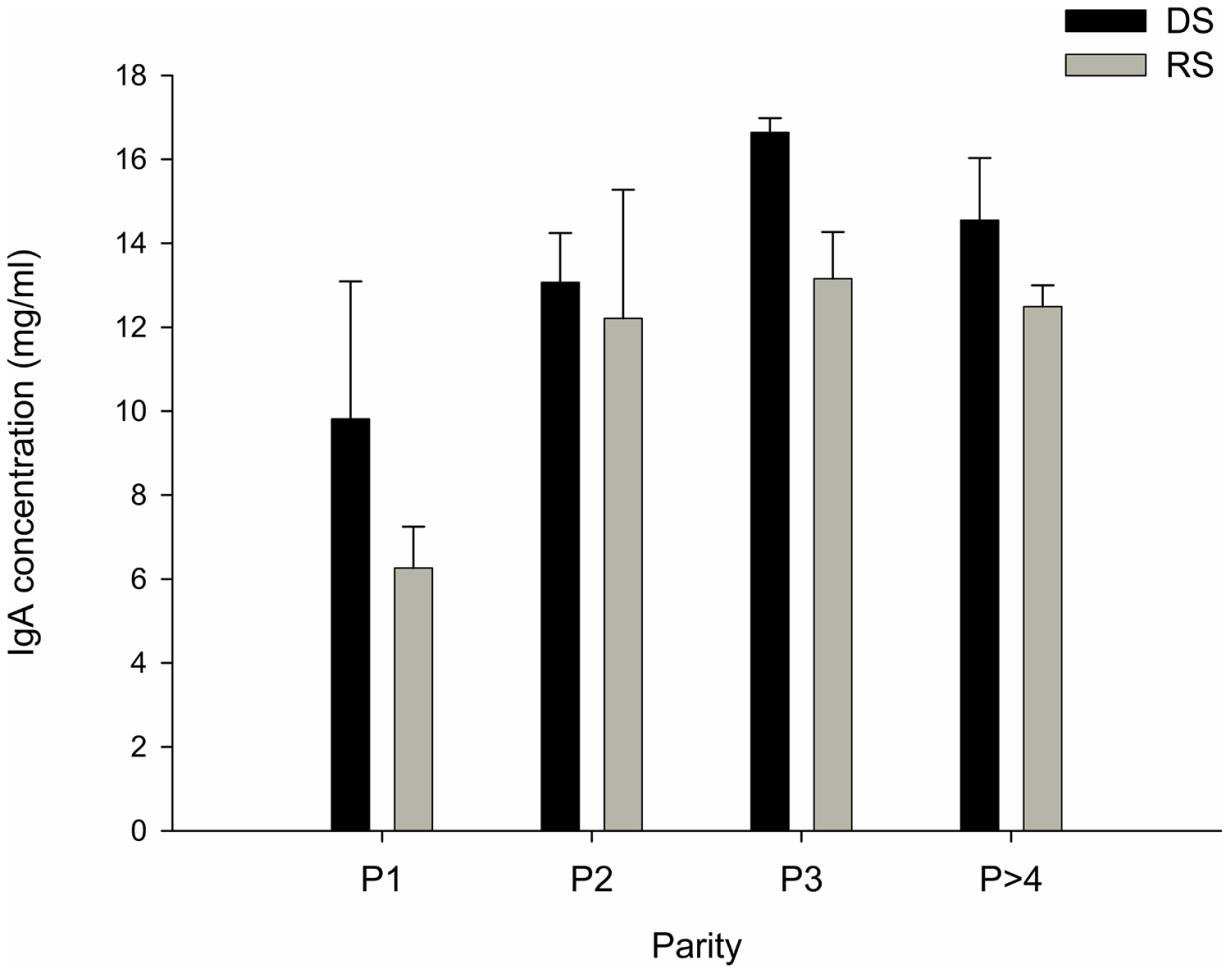
IgA concentration (mg/ml) in colostrum of sow. DS sows are represented with the black bar (N = 12) and RS sows with grey bars (N = 11) sows. Results are expressed as mean+SEM.

Immunoglobulin G, the most abundant Ig in colostrum, was not affected by the dietary treatment (55.2±4.17 mg/ml for DS group *vs* 52.1±2.69 mg/ml for the RS group, p = 0.80) but the parity tended (p = 0.06) to affect the IgG concentration, milk of P≥4 sows having higher IgG concentration than P1 and P3 sows (66.23±5.97 *vs* 48.93±4.41 and 48.58±2.21 mg/ml, respectively). IgA concentration in colostrum was not affected by the treatment (p = 0.07) but an effect of the parity was observed (p<0.01), IgA concentration being the lowest for the first parity (Fig 1).

### 3.3. Microbiota of sows’ faeces

**Fig 2:**
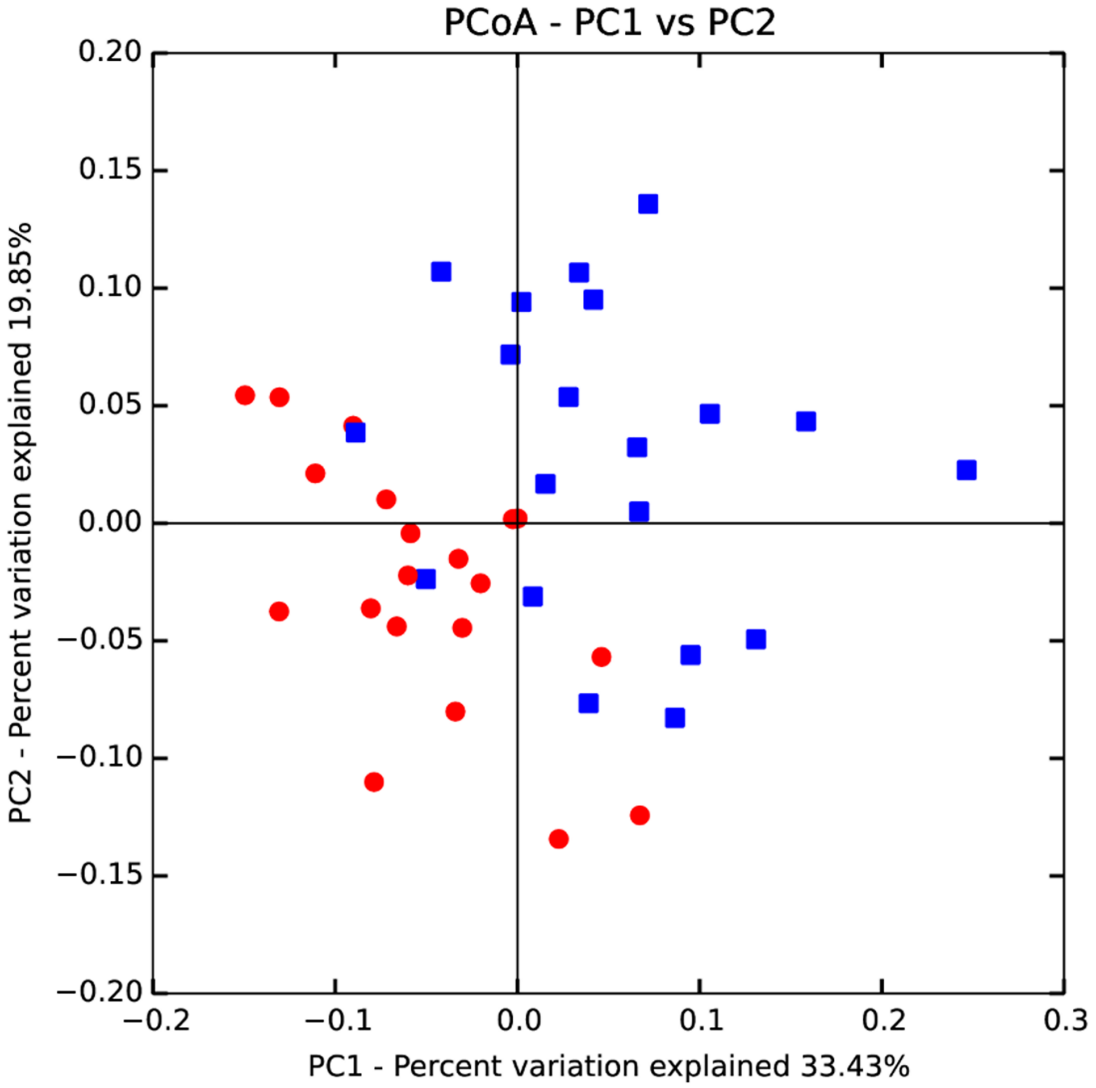
PCoA dicriminating periods. Individual red dots are the fecal samples of sows during gestation (N = 20) while blue squares are individual fecal samples of lactating sows (N = 20).

**Fig 3:**
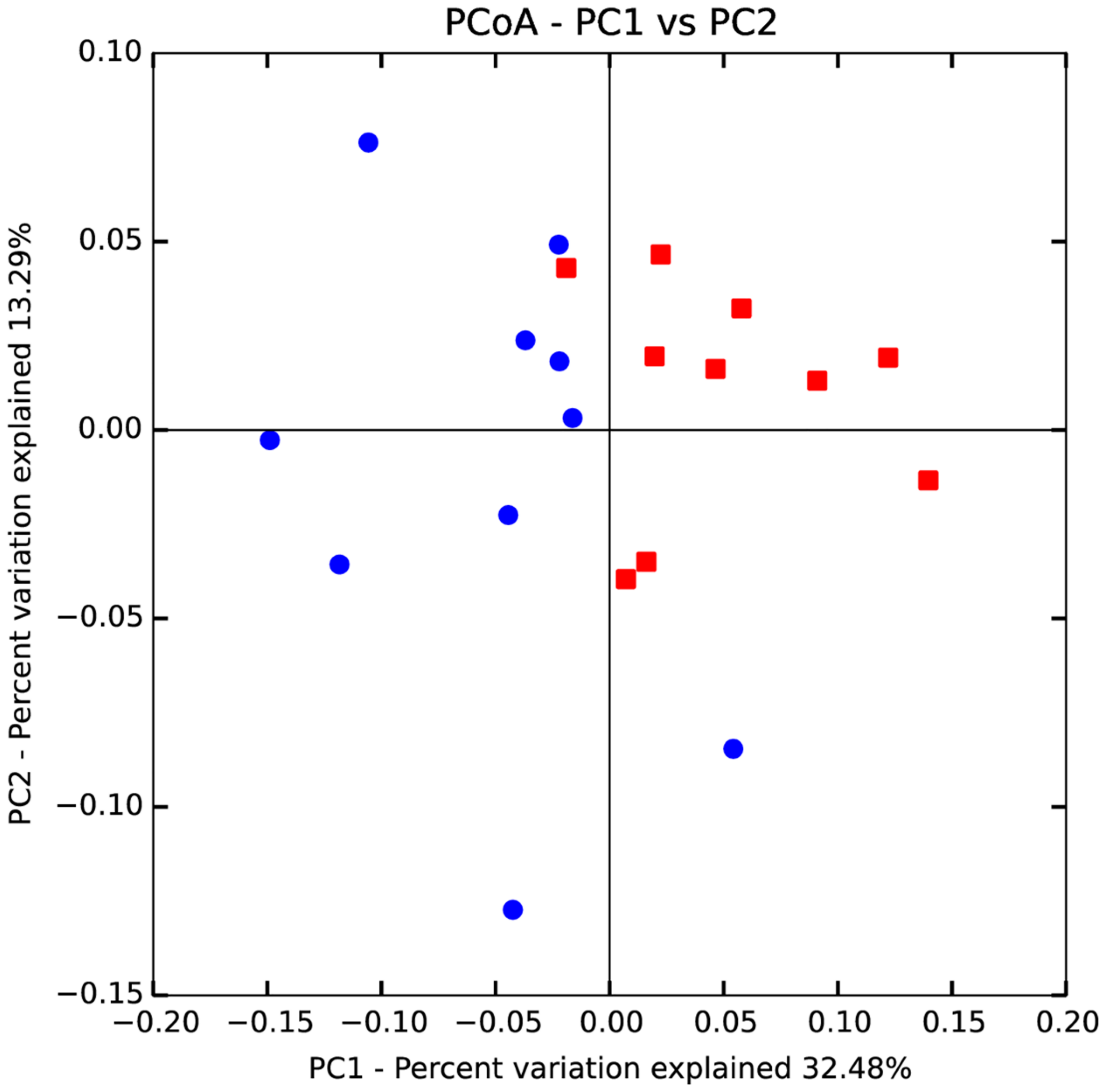
PCoA discriminating dietary treatments during gestation. Red squares represent the faecal microbiota composition of sows fed DS during gestation (N = 10) while blue dots represent microbiota of sows fed RS diet (N = 10).

Microbiota composition of the sows was determined during gestation and lactation; the effects of treatment and period were assessed. The number of observed OTUs and bacterial diversity (Shannon and Chao1 indexes) did not show any differences between treatments within each period. However, a different microbiota composition was observed between periods as seen by the PCoA discriminating gestation and lactation (Fig 2, PCoA based on the weighted Unifrac distance). Between gestation and lactation, a trend almost reached significance for a higher bacterial diversity during gestation as represented by the Shannon index (Gestation = 7.8±0.3 and lactation = 7.6±0.3, p = 0.06). Although bacterial diversity did not differ between treatments (Shannon index, P >0.10), the composition of the microbiota was affected during gestation as shown by the PCoA analysis (Fig 3). This clustering disappeared during the lactation period (S2 Fig). Statistical analyses for beta diversity showed the effect of the diet during gestation (p = 0.001) but no more during lactation (p = 0.56), while the parity did not seem to impact beta diversity, even though a trend was present (p = 0.09 during gestation and p = 0.07 during lactation).

The clustering between treatments during gestation translated differences in microbiota composition both at the phylum and genus levels. At the phylum level, the most abundant phyla during both periods for the two groups were *Firmicutes*, *Bacteroidetes* and *Spirochaetes*. Differences in microbial composition between treatments were observed during gestation as *Firmicutes* (p<0.01, FDR < 0.05) and *Euryarchaeota* (p<0.05, FDR = 0.05) proportions in the faecal microbiota of RS sows increased while *Bacteroidetes* (p<0.01, FDR<0.05), *Spirochaetes* (P<0.01, FDR<0.05) and *Tenericutes* (P<0.01, FDR = 0.17) relative abundances decreased compared to the DS treatment (Table 5). During lactation, only the minor Phylum *Lentisphaerae* (p<0.01, FDR = 0.12) proportion increased in the RS group compared to the DS group. The ratio *Firmicutes*:*Bacteroidetes* was impacted by the dietary treatment during gestation (1.59±0.07 for DS *vs* 2.11±0.15 for the RS sows, p = 0.005) while no effect of the dietary treatment was observed during lactation (2.36±0.32 for DS *vs* 2.43±0.17 for RS sows, p = 0.84).

**Table 5:** Relative abundances of the phyla and genera in sows’ faeces. Only genera present at >0.01% in the faecal microbiota of the sows fed either digestible starch (DS) or resistant starch (RS) -based diets during gestation and lactation were considered.

Genus	Gestation				SEM	Lactation				SEM
	DS (n = 10)	RS (n = 10)	P	FDR		DS (n = 10)	RS (n = 10)	P	FDR	
***Actinobacteria***	1.78	2.14	0.08	NS	0.22	1.88	1.90	NS	NS	0.25
*Bifidobacterium*	0.92	1.36	**0.02**	NS	0.21	1.18	1.15	NS	NS	0.23
***Bacteroidetes***	34.27	29.19	**<0.01**	**0.03**	0.96	28.69	27.08	NS	NS	1.13
*Prevotella*	12.11	8.80	NS	NS	0.90	10.32	10.39	NS	NS	0.91
Unclassified_*Bacteroidales*	10.75	8.88	NS	NS	0.62	8.54	7.00	NS	NS	0.58
Unclassified_S24-7	4.06	4.99	NS	NS	0.52	2.90	3.39	NS	NS	0.26
Unclassified_RF16	1.53	0.80	**0.01**	NS	0.16	0.98	0.91	NS	NS	0.11
Unclassified_p-2534-18B5	1.44	1.97	0.07	NS	0.16	1.30	1.39	NS	NS	0.16
CF231	1.29	0.90	0.07	NS	0.11	1.18	1.32	NS	NS	0.14
***Euryarchaeota***	0.17	0.51	0.05	0.05	0.07	0.16	0.20	NS	NS	0.03
Unclassified_R4-45B	0.15	0.12	NS	NS	0.01	0.05	0.13	**0.02**	NS	0.02
***Firmicutes***	53.25	59.86	**0.04**	**0.04**	1.15	62.24	63.70	NS	NS	1.27
Unclassified_*Ruminococcaceae*	17.75	20.68	**0.02**	NS	0.59	17.10	17.27	NS	NS	0.78
Unclassified_*Clostridiales* OTU1	7.76	8.54	NS	NS	0.28	7.21	8.17	NS	NS	0.28
Unclassified_*Lachnospiraceae*	3.71	3.48	NS	NS	0.15	3.92	3.28	0.06	NS	0.14
*Phascolarctobacterium*	2.90	2.52	NS	NS	0.22	2.24	2.15	NS	NS	0.12
Unclassified_*Christensenellaceae*	2.79	3.30	NS	NS	0.36	2.50	2.73	NS	NS	0.32
*Streptococcus*	2.74	2.03	NS	NS	0.83	1.58	3.37	NS	NS	0.58
*Oscillospira*	2.24	1.92	NS	NS	0.10	2.05	1.78	**0.04**	NS	0.07
*Lactobacillus*	2.03	2.73	NS	NS	0.42	13.11	10.35	NS	NS	1.50
Unclassified_*Clostridiaceae*	1.22	1.84	**0.02**	NS	0.11	1.59	2.07	**0.02**	NS	0.12
*Coprococcus*	0.83	1.22	**0.02**	NS	0.08	1.28	1.40	NS	NS	0.09
Unclassified_*Clostridiales* OTU2	0.49	0.79	**0.01**	NS	0.05	0.50	0.56	NS	NS	0.03
SMB53	0.49	0.79	**0.05**	NS	0.07	1.05	1.55	**<0.005**	NS	0.08
*Turicibacter*	0.33	1.10	**<0.005**	NS	0.12	0.65	1.29	**0.01**	NS	0.12
*Sharpea*	0.21	0.79	**0.03**	NS	0.15	0.05	0.11	NS	NS	0.03
*Methanobrevibacter*	0.12	0.46	**0.01**	NS	0.06	0.13	0.17	NS	NS	0.03
*Helicobacter*	0.12	0.06	**0.04**	NS	0.02	0.03	0.05	NS	NS	0.01
***Proteobacteria***	2.92	2.29	NS	NS	0.24	1.46	1.75	NS	NS	0.13
*Campylobacter*	0.97	0.67	0.07	NS	0.08	0.31	0.34	NS	NS	0.04
Unclassified_*Peptostreptococcaceae*	0.12	0.20	**0.02**	NS	0.01	0.23	0.33	**0.05**	NS	0.03
***Spirochaetes***	5.25	3.60	**0.03**	**<0.01**	0.28	3.96	3.50	NS	NS	0.26
*Treponema*	4.20	3.10	**0.01**	NS	0.23	3.25	2.72	NS	NS	0.22
*Sphaerochaeta*	1.05	0.50	**<0.005**	NS	0.10	0.71	0.78	NS	NS	0.08
***Tenericutes***	0.35	0.24	**0.04**	NS	0.03	0.38	0.35	NS	NS	0.03
*Anaeroplasma*	0.18	0.09	0.08	NS	0.02	0.13	0.12	NS	NS	0.01
L7A E11	0.11	0.19	0.07	NS	0.02	0.06	0.08	NS	NS	0.01

At the genus level (Table 5), the major differences in sows’ faecal microbiota composition between treatments also appeared during gestation. The most abundant genera were an unclassified *Ruminococcaceae*, *Prevotella*, unclassified *Bacteroidales* and *Clostridiales*, and *Treponema*. Within these major components of faecal microbiota, the unclassified *Ruminococcaceae* was increased (p<0.05) and *Treponema* was decreased (p<0.05) significantly during gestation in the RS group; this difference disappeared during lactation. Twelve other genera differed (p<0.05) between gestation while only 6 genera differed during lactation; the relative abundances of *Bifidobacterium*, *Coprococcus*, an Unclassified *Clostridiales* OTU2, *Sharpea*, *Methanobrevibacte*r and an unclassified *Peptostreptococcaceae* relative abundances were increased (p<0.05) in the faeces of sows fed RS during lactation. While *Oscillospira* decreased during lactation. An unclassified *Clostridiaceae*, SMB53 and *Turicibacter* increased both during gestation and lactation. It is worth noting that the proportion of *Lactobacilli* jumped from a mean of 2.38±0.42% during gestation to 11.73±1.50% during lactation, but this difference could not be attributed to the drop of one particular genus as the Pearson correlation analysis did not reveal absolute r-values higher than 0.6 (data not shown).

### 3.4. Microbiota of the colonic content of piglets at 26 days of age

The main abundant Phyla in the colon of piglets before weaning were *Firmicutes* (44.2±2.9%), *Bacteroidetes* (39.6±1.8%), *Proteobacteria* (6.9±1.0%) and *Fusobacteria* (5.7±1.9%). They were not impacted by the maternal dietary treatment, neither was the ratio between *Firmicutes* and *Bacteroidetes* (1.35±0.16 for DS piglets; 1.02±0.16 for RS piglets). At the genus level, the microbiota was mainly composed of *Prevotella*, unclassified *Ruminococcaceae*, *Lactobacillus* and *Bacteroides* (see Table 6). None of the 10 most abundant genera in the colon of the piglets were significantly affected by the maternal diet, while only genera present at a lower relative abundance than 1% of the total microbiota showed a trend (p<0.10), including *Veillonella*, unclassified *Clostridiales*, *Pasteurellaceae* and *Dethiosulfovibrionaceae* and *Brachyspira*.

**Table 6:** Relative abundances in piglets' colonic contents. Results showed are only for the top 10 genera and the genera with p<0.10 and relative abundance >0.01%.

Genus	DS (n = 7)	RS (n = 8)	P	FDR	SEM
***Bacteroidetes***	41.48	37.88	NS	NS	1.76
*Prevotella*	19.39	14.68	NS	NS	1.92
*Bacteroides*	5.44	8.25	NS	NS	1.24
Unclassified_*Bacteroidales* OTU1	4.51	4.86	NS	NS	0.54
Unclassified_S24-7	4.34	3.43	NS	NS	0.59
Unclassified_*Bacteroidales* OTU2	2.84	2.91	NS	NS	0.45
***Firmicutes***	42.73	45.42	NS	NS	2.88
Unclassified_*Ruminococcaceae*	12.19	13.43	NS	NS	1.38
*Lactobacillus*	5.55	3.94	NS	NS	1.02
Unclassified_*Clostridiales* OTU1	3.18	6.46	NS	NS	1.18
*Phascolarctobacterium*	2.95	3.71	NS	NS	0.25
*Oscillospira*	2.37	2.75	NS	NS	0.27
*Veillonella*	1.09	0.44	0.08	NS	0.20
Unclassified_*Clostridiales* OTU2	0.03	0.01	0.06	NS	0.00
***Fusobacteria***	4.91	6.37	NS	NS	1.94
*Fusobacterium*	4.91	6.37	NS	NS	1.94
***Proteobacteria***	7.89	6.12	NS	NS	1.03
Unclassified_*Enterobacteriaceae*	3.21	1.92	NS	NS	0.74
Unclassified_*Pasteurellaceae*	0.01	0.03	0.08	NS	0.01
***Spirochaetes***	0.75	1.46	NS	NS	0.33
*Brachyspira*	0.01	0.00	0.06	NS	0.00
***Synergistetes***	0.12	0.57	NS	NS	0.22
*Pyramidobacter*	0.11	0.55	NS	NS	0.21
Unclassified_*Dethiosulfovibrionaceae*	0.00	0.01	0.07	NS	0.00

### 3.5. SCFA, calprotectin concentration in digesta and gut morphology

Total content and molar ratios of individual SCFA and branched-chain fatty acids (BCFA) were not affected by the dietary treatment neither in the faeces of sows nor in the intestinal contents of piglets (S2 and S3 Tables). Calprotectin concentration in the colon of piglets did not differ (p = 0.85) either (39.03 ± 2.56 and 38.45 ± 1.58 pg/ml for piglets born from DS and RS sows, respectively). Maternal dietary treatment had no effect on villus height, crypt depth and the villi/crypts ratio (V:C), either in the duodenum, the jejunum or the ileum of the piglets (S4 Table).

### 3.6. Gene expression

In the ileum and colon of piglets, no differences were observed for cytokines involved in inflammatory processes, but an effect of the maternal diet was observed on tight junction protein expression. Indeed, *ZO-1* was more expressed in the ileum of piglets born from RS mothers (Table 7). *OCLN* tended to be more expressed in the ileum of RS piglets without reaching significance (p = 0.08).

**Table 7:** Relative gene expression in the ileum and colon of piglets at weaning. The 2^-ΔΔCt^ value of DS piglets is set at for each gene 1 to allow comparisons.

Gene	Ileum				Colon			
	DS (n = 7)	RS (n = 8)	SEM	P	DS (n = 8)	RS (n = 8)	SEM	P
*TNF-α*	1.00	0.91	0.07	NS	1.00	1.02	0.16	NS
*IL-6*	1.00	0.95	0.21	NS	1.00	2.55	1.04	NS
*NFκB*	1.00	0.99	0.03	NS	1.00	1.06	0.06	NS
*TGFβ*	1.00	0.91	0.04	NS	1.00	1.00	0.11	NS
*IFNγ*	1.00	0.56	0.17	NS	1.00	0.83	0.34	NS
*IL-1β*	1.00	0.84	0.21	NS	1.00	0.52	0.32	NS
*IL-10*	1.00	1.11	0.23	NS	1.00	2.08	0.66	NS
*ZO-1*	1.00	1.16	0.03	**0.02**	1.00	1.23	0.08	NS
*OCLN*	1.00	1.38	0.09	0.08	1.00	0.80	0.20	NS

### 3.7. Performances of piglets after weaning

The maternal treatment did not affect the ADG of the piglets (82.07±15.43g/day and 70.48±13.71g/day for the DS and RS piglets respectively during the first week post-weaning, 167.49±20.13g/day and 206.30±21.31g/day for the DS and RS pigs respectively during the second week post-weaning). Bodyweight of the piglets was not affected by the maternal dietary treatment either (6.71±0.31kg and 6.66±0.30kg for DS and RS pigs one week after weaning and 7.88±0.38kg and 8.35±0.36kg for DS and RS pigs 2 weeks after weaning, P for the treatment = 0.98).

**Fig 4:**
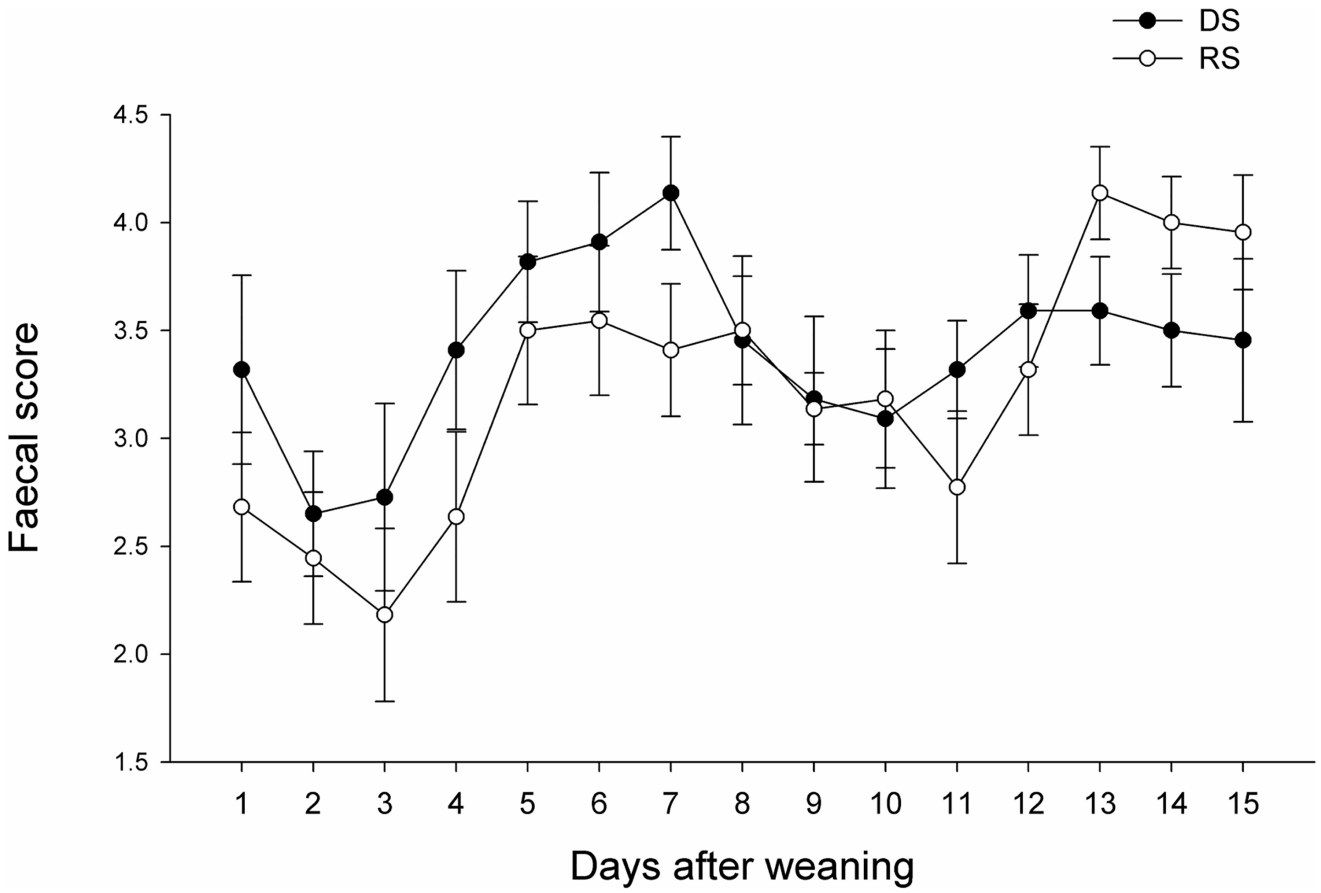
Piglets' faecal score during 2 weeks post-weaning. Score was assessed daily for 15 days.

A time effect (p<0.001) and an interaction between time and treatment (p = 0.005) was observed for the faecal scoring practised after weaning (Fig 4). On day 7, RS piglets had a lower score than DS piglets while they had a higher score on day 13, without reaching significance (p<0.10). The diarrhoea occurrence was calculated on 3-days intervals. The diarrhoea occurrence increased from period 1 to 2 and from period 4 to 5 (p<0.001, S3 Fig).

## Discussion

The main objective of this study was to investigate the maternal effect of a diet rich in pea starch as a source of RS on the intestinal microbiota and gut health-related parameters of the progeny. The hypothesis was that including resistant starch in the diet of the sows during gestation and lactation would favourably modify their milk and/or microbiota composition and that it would in turn affect piglets’ microbiota profile and their absorptive and immune abilities.

The unaffected growth performances of sows and piglets observed in our study is desirable as inclusion of RS, lower in energy content than its digestible counterpart, should not impair the performances of the animal. Yan *et al*. (2017) [19] fed sows high amylose maize (65% during gestation, 60% during lactation) and observed a lower birthweight for piglets born from high amylose sows. However, these piglets were able to catch up during lactation thanks to a higher fat content of the milk. In our study, no impact on the milk fat was observed. A reason for this discrepancy between the present study and Yan *et al*.’s (2017) [19] may reside in the fact that different breeds were used as breed can impact fat concentration [37] and that the amount of RS incorporation in the diet differed. In our study, parity as only factor did not affect the milk fat percentage but the interaction between parity and time was significant (p = 0.02), showing that the colostrum of gilts (parity 1) contained more fat than other parities.

Even though no difference in fat content was observed, other nutritional components of milk were affected by the sows’ diet. In particular, a decrease in protein concentration was observed for the RS sows compared to the DS group, together with a higher concentration of lactose in colostrum and milk collected one week after farrowing, while the opposite was observed during the last week of lactation. The lower milk protein concentration could be attributed to a slightly lower analysed protein content of the RS lactation diet. The discrepancy between our study and Loisel *et al*. (2013) [12], who did not observe any increase in lactose concentration in milk during the whole lactation period after feeding sows a high fibre gestation diet, can be explained by the fact that the type of supplementation given to sows differentially affects lactose concentration [38]. The increase in lactose concentration in RS milk in the beginning of lactation was probably too small to result in bodyweight difference for the piglets or to affect gut morphology. However, as the milk yield was not measured in this study, it cannot be excluded that DS sows had a higher milk yield, compensating the richer milk of RS sows. In the future, analysing the composition of milk oligosaccharides would be interesting, as oligosaccharides are considered as prebiotics, shaping the gut microbial communities of the piglets and are present in 29 forms in sows’ milk [39].

Microbiota results showed that in the faeces of the sows, more genera differed between dietary treatments during gestation than during lactation, as already observed by Leblois *et al*. (2017) [26] when feeding sows a high wheat bran diet. During gestation, even if the bacterial diversity and richness were not affected by the diet, we observed a clustering per dietary treatment on the PCoA graph that can be explained by differences both at the phylum and genus levels. Interestingly, during gestation, the RS group had a higher *Firmicutes* to *Bacteroidetes* ratio. As an increased *Firmicutes* proportion is usually related to a higher extraction of energy from the diet [39] and an increase in *Bacteroidetes* in humans has been associated with weight loss [40]; a higher ratio *Firmicutes*:*Bacteroidetes* would be thus desired in animal production. However, in the short term, the altered ratio observed during gestation did not lead to any bodyweight gain differences between the two groups of sows. In humans however, Martinez *et al*. (2010) [41] observed decreased abundance of *Firmicutes* and increased *Bacteroidetes*, hence a decreased *Firmicutes*:*Bacteroidetes* ratio, when adding chemically modified RS4 in the diet. Surprisingly, this was not observed for native RS starch granules (RS2) supplementation.

In agreement with the study of Sun et al. (2015) [42] who fed growing pigs raw potato starch and analysed the microbiota in the proximal colon, our study also showed an increase in the abundance of *Turicibacter* and *Coprococcus* and a decrease in Treponema and *Oscillospira* relative abundances in sows’ faeces. Interestingly, *Turicibacter* has been reported to be related to host gut immune status as this genus decreased or disappeared in immunodeficient animals [43]. In addition, the increase in the beneficial genus *Bifidobacterium* due to RS observed during gestation is in line with other studies [41,44]. Therefore, the increase in *Bifidobacterium* and *Turicibacter* observed in the current study migh suggest a better gut health. In contrast with Bird *et al*. (2007) [44] and Haenen *et al*. (2013) [17], no effect on *Lactobacillus* relative abundance in sows’ faeces was observed. Unfortunately, those microbial changes were not transferred to the offspring. It is noteworthy that effects and relative abundances of the genera differ between studies, the main reasons residing in the RS types used, breeds, environment, age, physiological stage, part of the gut studied, choice of hypervariable region for sequencing, and DNA extraction protocols/kits.

While a treatment effect was observed for the sow’s faecal microbiota during gestation, these differences disappeared during lactation. Even though ADF and NDF differences between DS and RS diets existed only during gestation, we assume that the microbiota difference is mainly due to the RS difference, for the following reasons. Firstly, RS is more extensively fermented than cellulose and hemicellulose (represented by ADF and NDF) [22]. Secondly, the observed genera-changes due to the RS treatment (for which ADF and NDF fractions were lower than in DS diet) during gestation are in line with other studies feeding pigs with RS [18,44] and are oriented to fermentative-type bacteria (increased *Firmicutes* and *Ruminococcaceae*). Therefore, we assume the observed changes during gestation can be attributed to the RS rather than the difference in hemicellulose and cellulose content.

The lack of differences in sow’s faecal microbiota between treatments during lactation is difficult to explain. However, this is in line with our previous study on wheat bran (Leblois et al., 2017) for which microbiota changes occurred during gestation when feeding sows diets containing the same ADF content but variable amounts of NDF (22% *vs* 25%) and wheat bran (0 *vs* 24%). Hence, it cannot be excluded that the hemicellulose difference existing during only gestation could as well have interfered with the absence of microbial changes during lactation. On the other hand, physiological and environmental changes that the sows face at farrowing and the stresses they encounter throughout the lactation period (manipulation of piglets, milking of the sows) might have such an impact on the microbiota that they can mask the effects of the dietary treatment. Indeed, Paβlack et al. (2015) [10] have already shown a more important time effect (gestation/farrowing/lactation) on the bacterial composition of sows’ faeces than inulin effect.

The microbiota changes occurring around farrowing and lactation were probably responsible for the absence of difference between the microbiota of piglets born from DS and RS sows. The similar microbiota between piglets was reflected by the same total and individual SCFA production in the caecum and colon of the piglets. Piglets’ microbiota composition considerably differed from the microbiota of the sows, which is in agreement with Leblois *et al*. (2017) [26] and resides in the fact that microbiota still did not reach a stable community that can only be achieved after weaning, maturation and introduction of solid feed. Moreover, piglets’ microbiota is not only acquired from bacteria present in sows’ faeces, but also from bacteria present in sows’ vaginal tract, milk and in the environment and is unstable in the neonatal gut until reaching a climax community with aging [45]. It can thus be suggested that changes induced in sows’ faecal microbiota induce limited alterations in the colonic microbiota of the piglet.

The immune competence of piglets relies both on the microbial colonization [14] and on the passive immunity acquired from the mother at birth via immunoglobulins transmitted in the colostrum [16]. Using seaweed extract, Leonard *et al*. (2011) [46] observed an increased concentration of IgG in colostrum of supplemented sows. In another study [12], feeding sows a high fibre diet during gestation decreased IgA concentration 24h after parturition. In our study, IgA concentration in colostrum of RS sows showed a trend (p = 0.07) for a decrease, while IgG concentration was not affected. A lower concentration of IgA in sows’ colostrum would be undesirable as IgA contributes to the passive immunity of the piglets.

The effect of the maternal supplementation with pea starch on the piglets was limited, as the milk composition was barely affected and as the microbiota of piglets was not affected by the maternal treatment. It is then likely that the performances and immune competence of the piglets remained unaffected as observed by similar litter bodyweight gains and percentage of weaned piglets for both treatments. As important as colostrum composition, the microbiota is crucial for the maturation of the gut immune system and has been shown to be the most important factor in the development of the intestinal immune system; moreover, different diets and environments inducing differences in microbiota have been shown to lead to differential immune cells development [47]. As no difference in microbiota composition was observed in those piglets raised in the same environment, it seems logical that the immune parameters of the piglets were not affected by the maternal treatment, as determined by the gene expression analysis. In contrast, Heim *et al*. (2015) [48] showed that seaweed-derived polysaccharides in the diet of gestating and lactating sows impacted the expression of inflammatory cytokines (higher expression of *IFNγ*, *IL-1*, *TGFβ1* and *TNFα* and lower expression of *IL-10* and *IL-6* in the ileum tissue) of piglets.

However, the protein *ZO-1* was more expressed in the ileum of RS piglets, while OCLN showed a trend (p = 0.08) for a higher expression. *ZO-1* is called a “plaque protein” and is involved in the strengthening of tight junction proteins by interacting with claudins that are involved in the closure of the gut membrane and also with junctional adhesion molecule Jam-A that is involved in the reduction of intestinal permeability [49]. A higher expression of *ZO-1* is thus beneficial for piglets as this will induce a stronger closure of the gut epithelium and a lower chance of translocation for pathogens, as tight junction proteins are responsible for paracellular permeability [36]. It is then likely than RS piglets would be less sensitive to pathogens at weaning, together with a lower passage for water loss causing diarrhoea. However, no significant differences on the faecal score or diarrhoea occurrence during the 2-weeks post-weaning period were observed for piglets born from DS and RS sows, and bodyweight gain or ADG were not affected either.

Thus, using pea starch in sows’ diets is not detrimental on piglets’ health but to obtain more conclusive results, it may be required to record faecal consistency for a longer period and to collect samples of intestinal tissues and contents further along during the post-weaning phase. In conclusion, the induced microbiota changes due to the diet of the sow did not affect the microbiota of piglets at weaning. However, milk composition can be affected by the inclusion of resistant starch in the diet of sows. Furthermore, the performances of the animals were not impacted by this supplementation and only minor effects of the tight junctions of piglets’ intestine were observed.

## Supporting information

S1 Table Bodyweight and backfat changes of the sows between periods. (XLSX)

S2 Table Total SCFA and molar ratios of acetate, propionate and butyrate in the faeces of sows. (XLSX)

S3 Table Total SCFA and molar ratios of individual SCFA and BCFA in piglets’ caecum and colon contents. (XLSX)

S4 Table Gut morphology (villus height, crypt depth, villus/crypt ratio) in the duodenum, jejunum and ileum of 26-days old piglets. (XLSX)

S1 Fig Piglets’ bodyweight from birth until weaning for maternal DS and RS treatments. (TIF)

S2 Fig PCoA discriminating dietary treatments during lactation. Red squares represent the faecal microbiota composition of sows fed DS during gestation (N = 10) while blue dots represent microbiota of sows fed RS diet (N = 10).
(TIF)

S3 Fig Diarrhoea occurence for weaned piglets during 15 days, divided in 3-days periods. (TIF)
