# Supplementary figures and images for: Feeding sows resistant starch during gestation and lactation impacts their faecal microbiota and milk composition but shows limited effects on their progeny
Source: PLoS One. 2018 Jul 3;13(7):e0199568. doi: 10.1371/journal.pone.0199568 (PMC6029764; doi:10.1371/journal.pone.0199568)

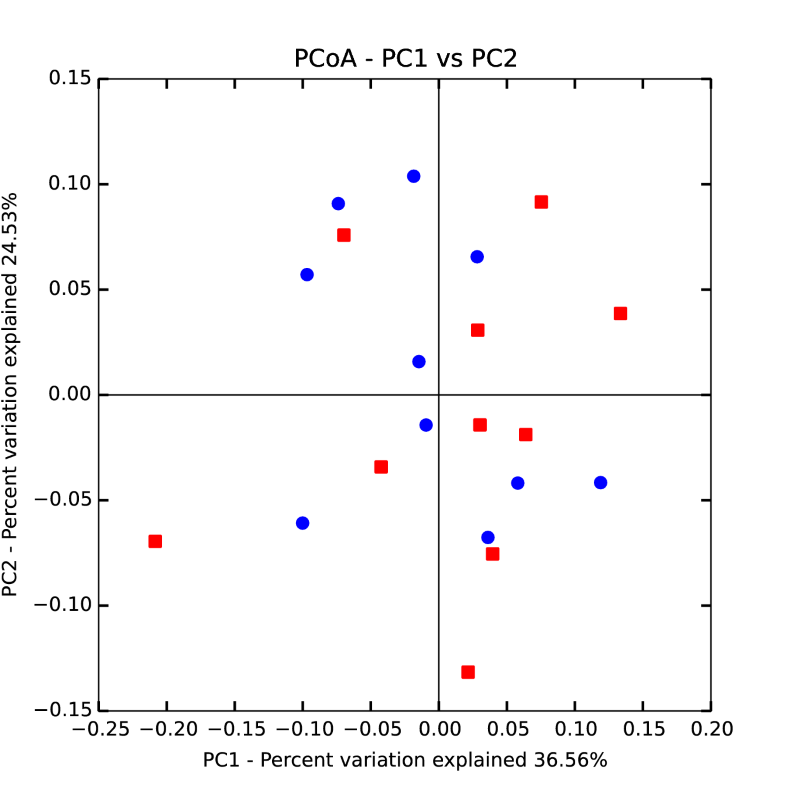

Supplement: S2 Fig — Red squares represent the faecal microbiota composition of sows fed DS during gestation (N = 10) while blue dots represent microbiota of sows fed RS diet (N = 10). (TIF) [file pone.0199568.s006.tif]
